# Impact of sex, fat distribution and initial body weight on oxytocin’s body weight regulation

**DOI:** 10.1038/s41598-017-09318-7

**Published:** 2017-08-17

**Authors:** Yuko Maejima, Masato Aoyama, Kazuho Sakamoto, Teruo Jojima, Yoshimasa Aso, Katsuya Takasu, Seiichi Takenosihita, Kenju Shimomura

**Affiliations:** 1 Department of Pharmacology, Fukushima Medical University School of Medicine, Fukushima-shi, 960-1295 Japan; 2 Department of Animal Science, Faculty of Agriculture, Utsunomiya University, Utsunomiya-Shi, 321-8505 Japan; 3 Department of Endocrinology and Metabolism, Dokkyo Medical University, Mibu-Machi, 321-0293 Japan; 4 Takasu Clinic, Nagoya-shi, 450-6208 Aichi Japan; 5 Advanced Clinical Research Center, Fukushima Global Medical Science Center, Fukushima Medical University, Fukushima-shi, 960-1295 Japan

## Abstract

Obesity is considered as a worldwide problem in both males and females. Although many studies have demonstrated the efficiency of oxytocin (Oxt) as an anti-obesity peptide, there is no comparative study of its effect in males and females. This study aims to determine factors (sex, initial body weight, and fat distribution) that may affect the ability of Oxt to regulate body weight (BW). With regard to sex, Oxt reduced BW similarly in males and females under both high fat diet (HFD) and standard chow-fed condition. The BW reduction induced by Oxt correlated with initial BW in male and female mice under HFD conditions. Oxt showed an equal efficacy in fat degradation in both the visceral and subcutaneous fat mass in both males and females fed with HFD. The effect of Oxt on BW reduction was attenuated in standard chow-fed male and female mice. Therefore, our results suggest that administration of Oxt is more effective in reducing BW in subjects with a high initial BW with increased fat accumulation. The present data contains important information for the possible clinical application of Oxt for the treatment of obesity.

## Introduction

The population prevalence of obesity (body mass index [BMI]≥30) is increasing throughout the world in both males and females^1^. Nowadays, obesity is one of the leading global risks for mortality. Recent articles have shown evidence that accumulation of visceral fat mass induces systemic inflammation and accelerates obesity, steatohepatitis, and arteriosclerosis.

Oxytocin (Oxt) is known as neural hormone that induces milk ejection and uterine contraction^2^ effects that are limited for females. However, Oxt is produced in the hypothalamus of both males and females and is released to other regions of the central nervous system or to whole body circulatory via posterior pituitary gland. Oxt receptors are abundantly distributed in several regions of the brain, including the cortical areas, olfactory systems, limbic systems, thalamus, hypothalamus, and brain stem^3^. There are no major differences in the distribution of Oxt receptors between male and female brains^3^. Within the latest decade, common functions of Oxt in both males and females have been revealed. Oxt is reported to increase trust in humans^4^, play a role in maternal behavior^5^, increase mother-infant bonding^6^, and improve social communication in autistic patients^7^.

As well as effects on social behaviour, Oxt is reported to play an important role in feeding regulation and energy metabolism in rodents, primates, and humans^8–13^. Intracerebroventricular or peripheral (intraperitoneal and subcutaneous) injection of Oxt decreases food intake in rats and mice^8, 9^. Oxt-knockout and Oxt receptor-knock out mice show low sympathetic tone and late-onset obesity^14, 15^. Subchronic (subcutaneous) treatment of Oxt increases energy expenditure, decreases body weight (BW) and fat mass, and improves steatohepatitis in rats, mice and monkeys^9, 11, 12, 16^. In addition, nasal administration of Oxt also decreases food or calorie intake in mice^17^ and humans^13^, and decreases BW in humans^12^.

Obesity is characterized as accumulation of subcutaneous and visceral fat. It has been shown that the ratio of visceral fat mass to total fat mass is lager in males, whereas the ratio of subcutaneous fat mass to total fat mass is larger in females^18^. Many articles have reported the effects of Oxt in treating obesity in rodents, primates, and humans^8–13, 16, 17^. However, such reports used only males or mixed data and, therefore, there are no studies comparing the effect of Oxt on BW regulation between males and females. Furthermore, no systematic studies have been reported comparing the effect of Oxt in male and female, high fat diet (HFD) and normal chow-fed mice. In order to determine the optimal condition for Oxt to treat obesity, such investigation is necessary.

Here we report a comparison of the effect of Oxt in males and females, and its effect on initial BW and fat distribution by using CT.

## Results

### The effects of Oxt on BW and food intake in HFD-fed male and female mice

**Figure 1:**
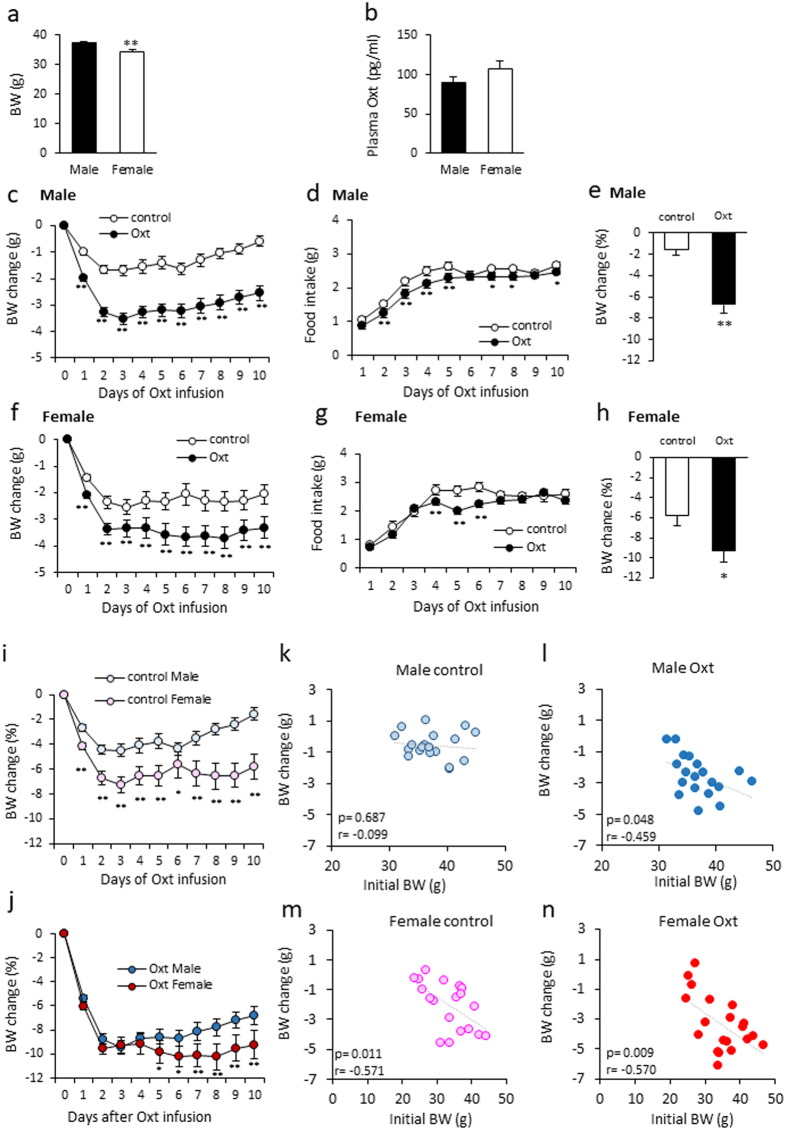
The effect of Oxt treatment on BW and food intake in male and female mice fed a high-fat diet. (**a**) Initial body weight of males and females used in this study. (**b**) Plasma Oxt levels in HFD-fed male and female mice (n = 8, 7). (**c**,**d**) Daily BW change (**c**) and food intake (**d**) during the Oxt infusion period in HFD-fed male mice. (n = 19, 19) (**e**) The percentage of BW change at 10 days after starting Oxt infusion in male mice. **P < 0.01 by unpaired Student’s *t*-test. (**f,g**) Daily BW change (**f**) and food intake (**g**) during the Oxt infusion in HFD-fed female mice. (n = 19, 20) *P < 0.05, **P < 0.01 by two-way ANOVA followed by Tukey’s multiple range test. (**h**) The percentage of BW change at 10 days after starting Oxt infusion in female mice. *P < 0.05 by unpaired Student’s *t*-test. (**i**) The percentage of BW change during 10 days in control male and female. *P < 0.05, **P < 0.01 by two-way ANOVA followed by Tukey’s multiple range test. (**j**) The percentage of BW change during 10 days in Oxt treated male and female. *P < 0.05, **P < 0.01 by two-way ANOVA followed by Tukey’s multiple range test. (**k,l**)The correlation of BW change at 10 days and initial BW (BW at day 0) in control (**k**) and Oxt-treated (**l**) male mice. (**m**,**n**) The correlation of BW change at 10 days and initial BW (BW at day 0) in control (**m**) and Oxt-treated (**n**) female mice.

The initial BW of the animals used in this study was 37.36 ± 0.64 g in males (n = 38) and 34.20 ± 0.99 g in females (n = 39) (Fig. 1a). The difference in BW was statistically significant. The plasma oxytocin levels are slightly high in female (106.67 ± 10.78 pg/ml) than male mice (89.75 ± 6.92 pg/mg), but there was no statistically significant difference (P = 0.198) (Fig. 1b).

In male mice, Oxt infusion rapidly decreased BW within one day and maximal BW loss was detected at 3 days after starting infusion (Fig. 1c). BW was maintained at a significantly reduced level during the Oxt infusion period compared to control (saline infused) mice (F_1,360_ = 1090.34, P < 0.01; Fig. 1c). Food intake was slightly, but significantly, less in Oxt infused mice compared to saline infused control mice (F_1,324_ = 47.66, P < 0.01; Fig. 1d). At day 10 after starting Oxt infusion, the percentage of BW reduction from the initial BW was significantly different from that of the saline-infused controls. (−1.55 ± 0.52% for control and −6.75 ± 0.76% for Oxt infused group; Fig. 1e).

On the other hand, in female mice, the pump implantation operation itself induced a reduction of BW (Fig. 1f). However, the BW reduction was slightly, but significantly, larger in the Oxt infused group compared to the control group during the Oxt infusion period (F_1,370_ = 205.72, P < 0.01; Fig. 1f). Oxt infusion also rapidly decreased BW change within 2 days after initiation and the change was maintained at the reduced level during the Oxt infusion period. Also, Oxt infused female mice showed significantly less food intake compared to the saline infused control female mice (F_1,333_ = 23.37, P < 0.01; Fig. 1g). The percentage of BW change at day 10 after initiating Oxt infusion was significantly decreased in female controls. (−5.80 ± 1.02% for controls and −9.25 ± 1.18% for Oxt infusion group) (Fig. 1h).

In comparing the percentage of BW changes from original BW (BW at day 0), the percentage reduction of BW in control female was significantly larger than that of control male mice (F_1,360_ = 247.4, P < 0.01; Fig. 1i). The percentages of BW change from original BW in Oxt treated female mice were slightly larger than that of Oxt treated male mice (F_1,370_ = 50.44, P < 0.01; Fig. 1j).

These results indicate that Oxt similarly induces reduction of BW in both male and female mice.

The correlation between initial BW and BW change at day 10 after starting Oxt infusion in male (Fig. 1k,l) and female (Fig. 1m,n) mice was analysed. In the male control mice, there was no correlation between BW change and initial BW (r = −0.099, p = 0.687) (Fig. 1k). On the other hand, a significant negative correlation was observed between initial BW and BW change in the Oxt infusion group (r = −0.459, p = 0.048) (Fig. 1l).

Surprisingly, a significant negative correlation between BW change and initial BW was detected in both the control (Fig. 1m; r = −0.571, p = 0.011) and Oxt infusion groups (Fig. 1n; r = −0.570, p = 0.009) in female mice.

### The effect of Oxt on fat distribution (visceral and subcutaneous fat) in male and female mice

**Figure 2:**
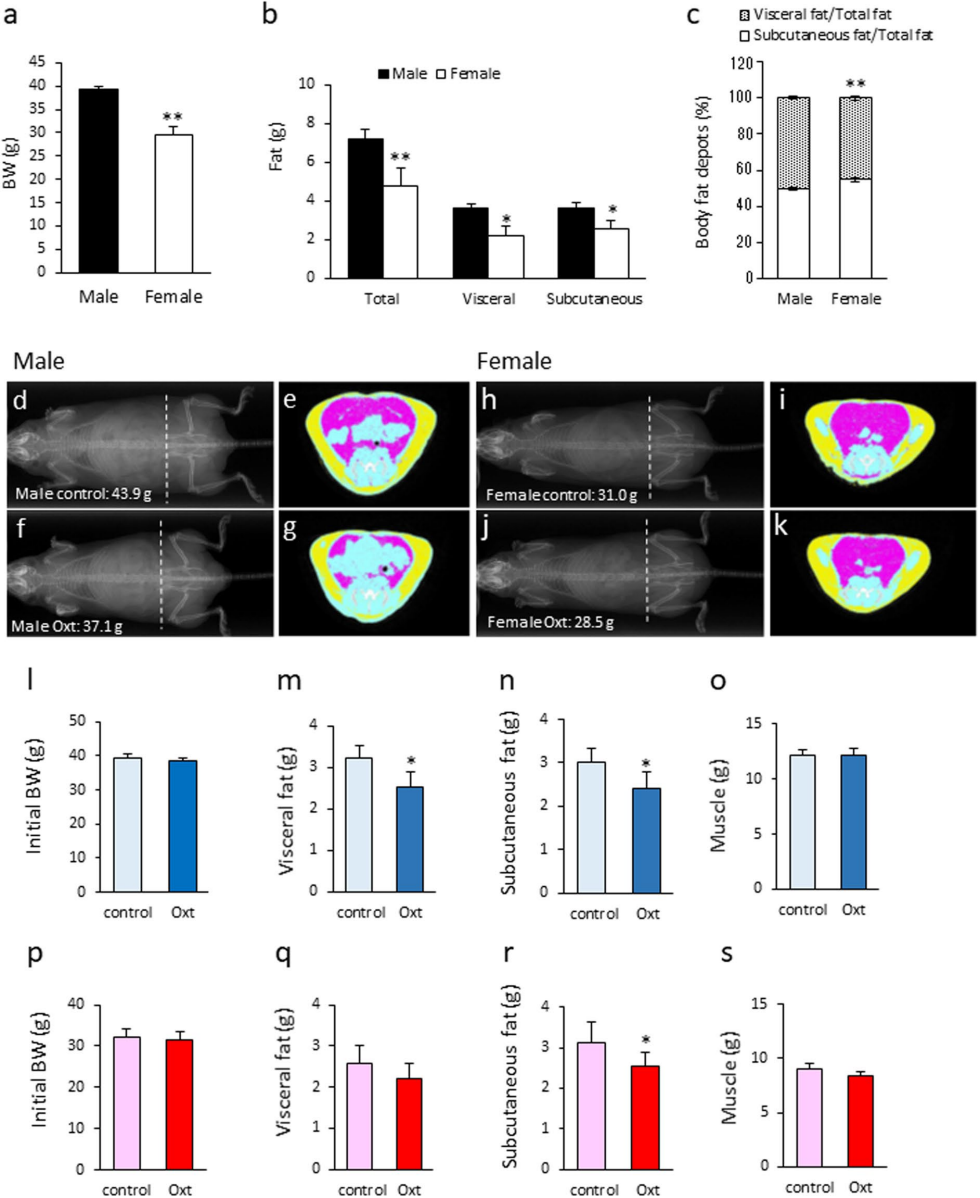
The effect of Oxt treatment on visceral fat and subcutaneous fat in male and female mice fed a high-fat diet. (**a**) Body weight of 8-week-old HFD-fed males and 12-week-old HFD-fed females. (n = 11, 9) (**b**) Weight of total fat, visceral fat, and subcutaneous fat analysed by CT scan in HFD-fed male and female mice. (n = 11, 9) (**c**) Ratio of the visceral fat to the total fat, and ratio of the subcutaneous fat to the total fat in HFD-fed male and female mice. (male n = 11, female n = 9). *P < 0.05, **P < 0.01 by unpaired Student’s *t*-test. (**d–g**) Representative X-ray (**d,f**) and CT image (**e,g**) of HFD-fed control (**d,e**) and Oxt-treated (**f,g**) mice. CT images of e and g are sectional images of the white dotted lines (level of vertebra L6) in (**d** and **f**), respectively. Yellow areas indicate subcutaneous fat and pink areas indicate visceral fat. The BW of mice scanned by CT was 43.9 g and 37.1 g in control and Oxt-treated mice, respectively. CT scanning was performed at 10 days after starting Oxt infusion. (**h–k**) Representative X-ray (**h,j**) and CT image (**i,k**) of HFD-fed control (**h,i**) and Oxt-treated (**j,k**) female mice. CT images of i and k are sectional images of the white dotted lines (level of vertebra L6) in (**h** and **j**), respectively. Yellow areas indicate subcutaneous fat and pink areas indicate visceral fat. The BW of mice scanned by CT was 31.0 g and 28.5 g in the control and Oxt-treated mice, respectively. CT scanning was performed at 10 days after Oxt infusion. (**l**) Initial body weight of the control and Oxt-treated group in HFD-fed male mice (n = 6 each). (**m–o**)The volume of visceral fat (**m**) and subcutaneous fat (**n**), and muscle (**o**) after Oxt infusion in male mice. (**p**) Initial body weight of the control and Oxt-treated group in HFD-fed female mice (n = 9 each). (**q–s**) The volume of visceral fat (**q**) and subcutaneous (**r**), and muscle (**s**) after Oxt infusion in female mice. *P < 0.05 by paired *t*-test. (n = 6).

In order to compare BW and fat distribution between male and female mice after HFD, CT analysis was performed. The BW of female mice fed with a high fat diet (HFD) for 12 weeks was significantly lower than that of male mice, which were fed with a HFD diet for 8 weeks (Fig. 2a). The weight of total fat mass, visceral fat mass, and subcutaneous fat mass were significantly lower in female mice compared with male mice (Fig. 2b). The ratio of visceral fat mass to total fat mass was significantly larger in male than in female mice (Fig. 2c). On the other hand, the ratio of subcutaneous fat mass to total fat mass was significantly larger in female than male mice (Fig. 2c).

At day 10 after initiating Oxt infusion, the distribution of fat (visceral and subcutaneous fat) was also analysed by using CT (Fig. 2d–k). Since a variance of BW is large, pairs of mice with similar BW were employed for this experiment. There was no significant difference in initial BW among male (Fig. 2l) and female (Fig. 2p) mice. As shown in the representative CT images for male mice in Fig. 2d–g, both visceral and subcutaneous fat were significantly decreased in the Oxt infusion group compared with the control group (Fig. 2m,n). The loss of each visceral and subcutaneous fat following Oxt infusion was approximately 20% (Fig. 2m,n). There were no significant differences in muscle volume between control and Oxt treated group (Fig. 2o).

As shown in the representative CT image for female mice in Fig. 2h–k, the visceral fat in the Oxt infused group tended to decrease (approximately 15% compared with the control group) (Fig. 2q). The amount of subcutaneous fat in the Oxt infused group was significantly decreased (approximately 20% reduction compared with that of the control group) (Fig. 2r). However, there were no significant differences in muscle between control and Oxt infused group (Fig. 2s).

From these results, it is clear that there is no specificity of Oxt’s effect between visceral and subcutaneous fat in male mice. However, in female mice, the effect of Oxt on fat degradation is slightly significant in subcutaneous fat than visceral fat. Similar to BW, Oxt similarly reduced fat mass in male and female mice.

### The effect of Oxt on BW in standard diet-fed male and female mice

The effect of Oxt on BW reduction was more potent when the initial BW was larger in HFD-fed mice.

Therefore, in order to examine the impact of initial BW on the effect of Oxt, BW and food intake after Oxt infusion in standard diet-fed male and female mice were examined.

**Figure 3:**
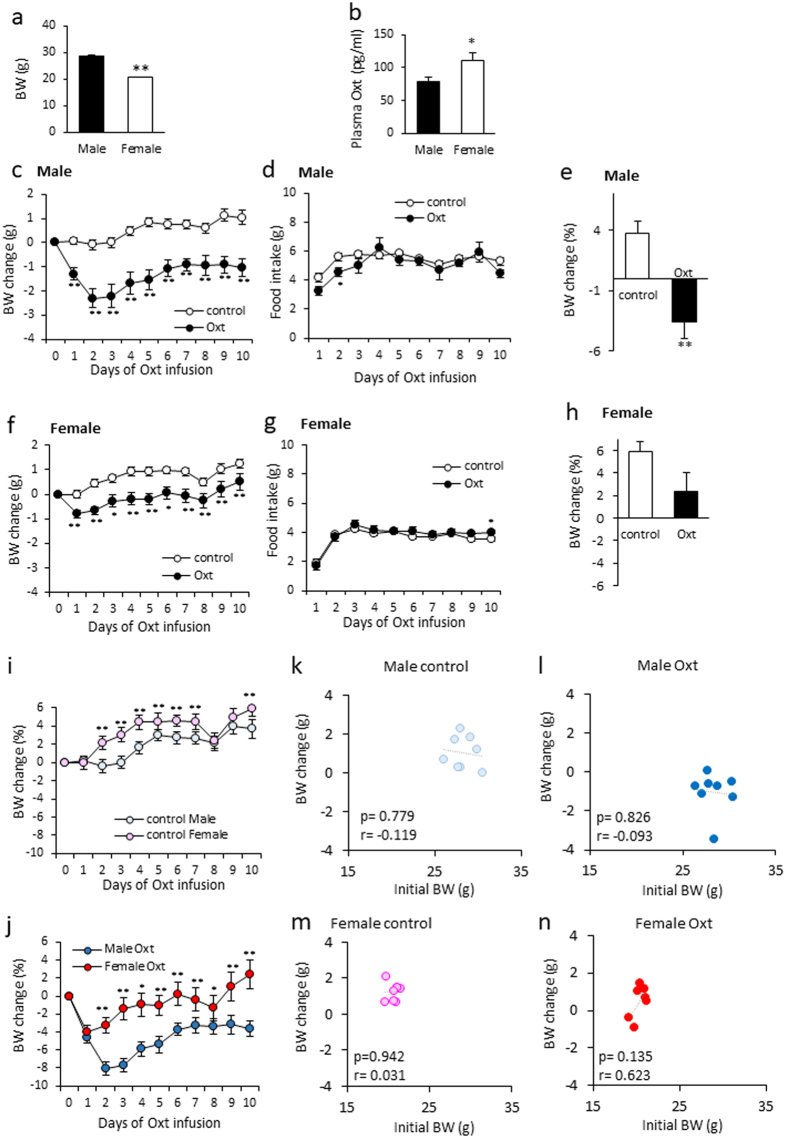
The effect of Oxt treatment on BW and food intake in male and female mice fed a standard-chow diet. (**a**) Initial body weight of males and females used in this study. (**b**) Plasma Oxt levels in standard chow-fed male and female mice (n = 10 each). (**c**,**d**) Daily change of BW change (**c**) and food intake (**d**) during the infusion period in standard chow-fed male mice. (n = 8 each). (**e**) The percentage of BW change at 10 days after starting Oxt infusion in male mice. **P < 0.01 by unpaired Student’s *t*-test. (**f**,**g)** Daily change of BW change (**f**) and food intake (**g**) during the infusion period in standard chow-fed female mice. (n = 7, 8) *P < 0.05, **P < 0.01 by two-way ANOVA followed by Tukey’s multiple range test. (**h**) The percentage of BW change at 10 days after starting Oxt infusion in male mice. P = 0.068 by unpaired Student’s *t*-test. (**i**) The percentage of BW change during 10 days in control male and female. **P < 0.01 by two-way ANOVA followed by Tukey’s multiple range test. (**j**) The percentage of BW change during 10 days in Oxt treated male and female. *P < 0.05, **P < 0.01 by two-way ANOVA followed by Tukey’s multiple range test. (**k**,**l**) The correlation of BW change at 10 days and initial BW (BW at day0) in the control (**k**) and Oxt-treated (**l**) male mice. (**m**,**n**) The correlation of BW change at 10 days and initial BW (BW at day0) in the control (**m**) and Oxt-treated (**n**) female mice.

The initial BW of mice used in this study was 28.33 ± 0.35 g (n = 16) and 20.50 ± 0.18 g (n = 15) in male and female, respectively. The difference in BW were statistically significant (Fig. 3a). The plasma oxytocin levels were significantly higher in female (110.50 ± 12.58 pg/ml) than male mice (78.63 ± 6.77 pg/mg) (Fig. 3b).

In the standard diet-fed male mice, daily BW change from the initial BW was significantly decreased within one day after starting the Oxt treatment (F_1,140_ = 441.57, P < 0.01; Fig. 3c). This significant loss of BW change was maintained throughout the period of Oxt infusion. The amount of food intake slightly but significantly decreased in Oxt-treated standard diet-fed male mice at day 2 (F_1,126_ = 6.56, P < 0.05; Fig. 3d). The percentage of BW change at 10 days compared with initial BW was significantly low in Oxt-treated standard diet-fed male mice (control: 3.71 ± 1.06%, Oxt: −3.6 ± 1.31%) (Fig. 3e).

In the standard diet-fed female mice, daily BW change from the initial BW was also immediately decreased after starting the Oxt treatment (F_1,130_ = 303.68, P < 0.01; Fig. 3f), and food intake was almost no differences between control and Oxt group (F_1,117_ = 5.03, P < 0.05; Fig. 3g). The percentage of BW change at 10 days compared with initial BW was nearly significantly different between the control group (5.93 ± 0.88%) and the Oxt group (2.38 ± 1.62%) (P = 0.068) (Fig. 3h).

In comparing the percentage of BW changes from original BW, the percentage change of BW was significantly larger in control female than that of male mice (F_1,140_ = 92.70, P < 0.01; Fig. 3i). The percentage of BW change from original BW in Oxt treated female mice were also significantly larger than that of Oxt treated male mice (F_1,130_ = 131.58, P < 0.01; Fig. 3j).

The correlation between initial BW and BW change at 10 days after starting Oxt infusion in standard diet-fed male (Fig. 3k,l) and female (Fig. 3m,n) mice was analysed. In the male control and Oxt-treated mice, there was no correlation between BW change and initial BW (control: r = −0.119, p = 0.779, Oxt: r = −0.093, p = 0.826) (Fig. 3k,l). Similar with the standard diet-fed male mice, there was no correlation between BW change and initial BW in standard diet-fed female mice (control: r = 0.031, p = 0.942, Oxt: r = 0.623, p = 0.135) (Fig. 3m,n).

### The effect of different dose of Oxt in HFD-fed mice

In order to examine the doe-dependent effect of Oxt on BW change and food intake, the osmotic mini pump containing saline, 800 and 1600 μg/kg/day dose of Oxt were prepared and daily BW change and food intake in HFD-fed male and female mice were measured.

**Figure 4:**
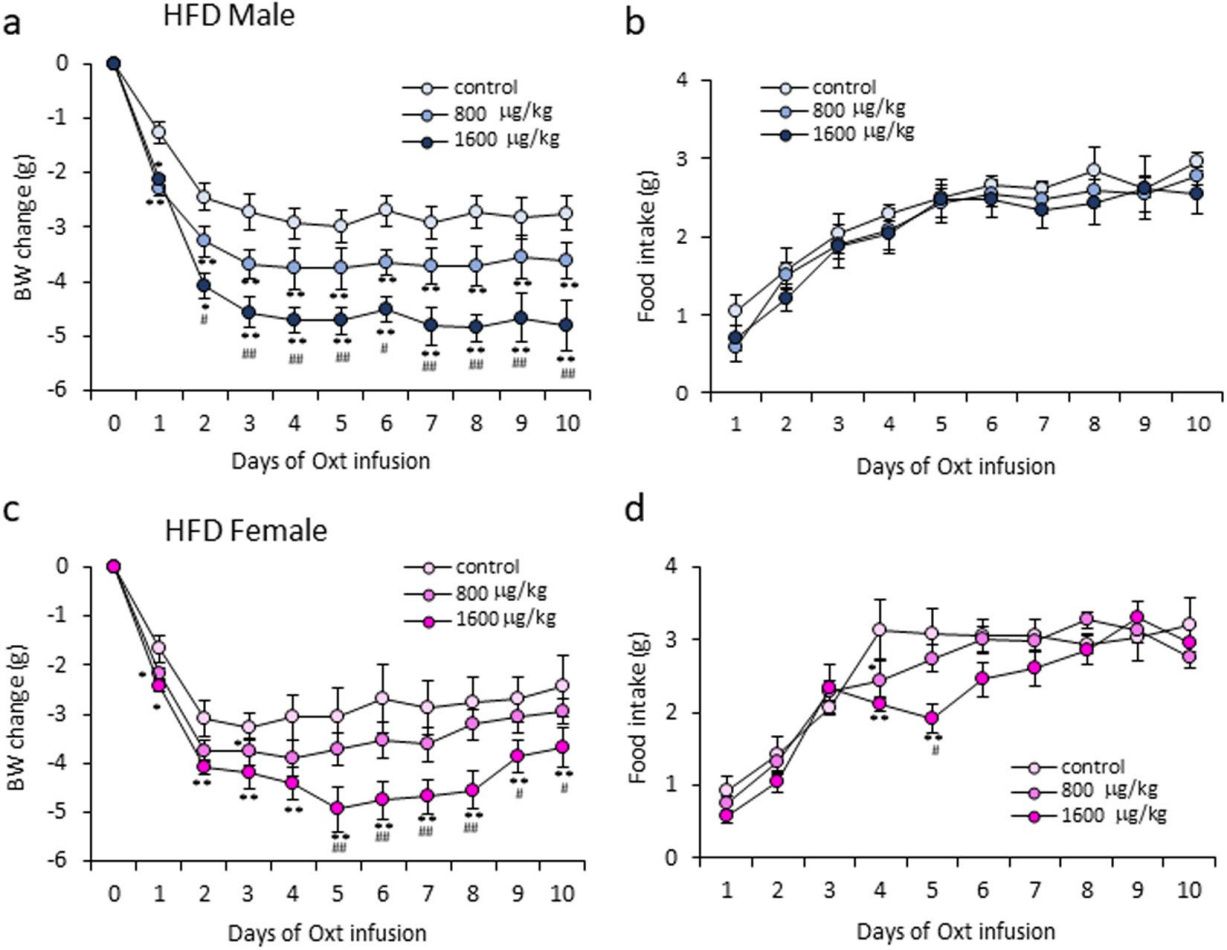
The different dose of Oxt treatment on BW and food intake in male and female mice fed a high fat diet. (**a**,**b**) Daily BW change (**a**) and food intake (**b**) during the different dose of Oxt (800 and 1600 μg/kg/day) infusion in HFD-fed male mice. (n = 6, 5, 5) **P < 0.01 by two-way ANOVA followed by Tukey’s multiple range test. (**c**,**d**) Daily BW change (**c**) and food intake (**d**) during the different dose of Oxt (800 and 1600 μg/kg/day) infusion in HFD-fed female mice. (n = 6, 7, 7) **P < 0.01 by two-way ANOVA followed by Tukey’s multiple range test.

In male mice, as shown in Fig. 4a, Oxt clearly decreased BW change dose dependently. However, dose dependent effect of Oxt on food intake was not detected (Fig. 4b).

In female mice, Oxt decreased both BW change (Fig. 4c) and food intake (Fig. 4d) dose dependently.

## Discussion

This study was designed to investigate the impact of Oxt on various factors that may affect BW regulation. Considering the negative correlation between the initial BW and BW change induced by Oxt infusion in HFD-fed mice and the less but clear effect of Oxt on BW in standard chow-fed mice, the effect of Oxt on BW change is depended on the original BW and, is similar in both males and females.

The interpretation of Oxt’s effect on female mice shown in this study is complicated. The variation of the effect of Oxt on BW was substantially large in HFD-fed female mice. One possible factor for this large variance could be the oestrous cycle. The levels of plasma ovarian hormones, such as estrogen and progesterone are different in each day, and these hormones are known to affect feeding and energy metabolism^19–22^. Also, the application of HFD can change the estrogen levels and induce acyclicity in mice^23^. Furthermore, estrogen increases expression of Oxt^24^ and Oxt receptor^25^. It raises the possibility that difference in estrogen levels based on oestrous cycle may have influenced the sensitivity of Oxt.

Another factor could be the influence of anaesthesia. We examined the effect of anaesthesia on BW in male and female mice fed with HFD and standard chow. Seven days after injection of anaesthesia, BW was significantly decreased in only HFD-fed males and females. However, this effect was significantly greater in the females than males (Male: −5.33 ± 0.90% (n = 6), Female: −10.59 ± 0.61% (n = 5)). Therefore, the factors “HFD” and “female” could have been affected by the usage of tribromoethanol. However, the use of anaesthesia was inevitable for the current experiments. This may well be a limitation of this study. Seven days after use of isoflurane, BW in HFD-fed female mice (n = 3) was increased 1.07 ± 0.86%. Thus, isoflurane could be considered as more appropriate anaesthesia for the experiments using HFD-fed obese model mice. In addition, HFD-fed male and female mice failed to recover from post-operation BW reduction. However, BW in standard chow-fed male and female control mice showed complete recovery at 10 days after surgery. These differences may be related to plasma glucose concentration. The fasting plasma glucose levels in HFD-fed mice were 179.6 ± 24.48 and 157.95 ± 20.23 mg/dl in males (n = 10) and females (n = 19), respectively. On the other hand, the fasting plasma glucose levels in standard chow-fed mice were 126.0 ± 13.21 and 122.63 ± 13.16 mg/dl in males (n = 11) and females (n = 8), respectively. Wound healing in diabetic patients is delayed in humans^26^ and high blood glucose is known to contribute to poor healing and ulcer formation^27^. Therefore, our current data cannot exclude the influence of high blood glucose level. However, the fact that Oxt was effective in HFD-fed mice indicates the effect of Oxt on obese subjects with high blood glucose. This is potentially important clinical information for type 2 diabetes patients since Oxt could improve insulin secretion^17^ and glucose tolerance^9^. Further studies are required to examine the pure effect of Oxt on BW reduction in female mice.

When comparing the effects of Oxt on food intake in HFD-fed male and female mice, a reduction was observed in both the males and females. This is consistent with a study by Benelli *et al*. reporting no sex differences in Oxt-induced inhibition of feeding^28^. On the other hand, recent articles have reported that Oxt’s inhibitory effect on food intake is stronger in obese than normal-weight men^29^. In this study, the effect of Oxt on feeding was slightly larger in HFD-fed mice than standard chow-fed mice in males and females. The expression levels of Oxt and Oxt receptors are reported to be increased in the brain of HFD-fed mice^30^. This mechanism concerning food intake may be related to the change of Oxt and Oxt receptor expression following development of obesity. Also, the pattern of food intake was different in our study. In male mice, food intake was decreased within the first half of the Oxt infusion period, consistent with our previous report^9^, but female mice showed a reduction of food intake for only three days in the middle of the experimental period. This difference may also arise from recovery following the anaesthesia and pump-infusion surgery.

Both subcutaneous and visceral fat mass was lower by 20% in Oxt-treated male mice. Similar to male mice, subcutaneous fat mass was significantly lower by 20% in Oxt-treated female mice compared with the control mice. However, there was no significant reduction of visceral fat mass in Oxt-treated female mice (P = 0.54). These differences may be due to the fat distribution in the female mice. In HFD-fed female mice, visceral fat mass was significantly less than subcutaneous fat mass (visceral fat: 2.22 ± 0.44 g, subcutaneous fat: 2.57 ± 0.44 g, P < 0.01, paired *t*-test), whereas in HFD-fed male mice, visceral fat mass and subcutaneous fat mass were at the same level (visceral fat: 3.63 ± 0.27 g, subcutaneous fat: 3.60 ± 0.20 g, P = 0.80, paired *t*-test).

These data suggested that the difference in Oxt’s effect on fat mass between male and female mice is due to the sex dependent difference in visceral and subcutaneous fat composition.

We also examined the chronic effects of Oxt on BW and food intake in standard diet-fed mice. In standard chow-fed male mice, BW at day 10 after starting Oxt infusion was decreased by 3.6%. Compared with BW reduction in HFD-fed male mice (6.7% decline), the effect of Oxt on standard diet-fed male mice was significantly attenuated (p = 0.04, unpaired *t*-test). Surprisingly, BW at 10 days after starting Oxt infusion in standard chow fed female mice was increased by 2% from the initial BW. Compared with BW reduction in Oxt infused HFD-fed female mice (−9.4%), the effect of Oxt on standard diet-fed female mice was significantly attenuated (p = 0.0004, unpaired *t*-test).

In this study, we hypothesized that the effect of Oxt on BW reduction depends on the initial BW and fat mass. A clinical study by Zhang *et al*. also reported that Oxt nasal treatment had more impact on BW reduction in individuals with a higher BW^12^.

It is known that Oxt receptors are expressed in adipocytes^31^, and activation of these receptors induces lipolysis and fatty acid β-oxidation^16^. As shown in Fig. 2l–s, Oxt decreased only fat mass but did not decrease lean mass (muscle). Consistent with our results, Altrriba *et al*. reported that Oxt treatment in ob/ob mice led to a fat mass loss without any change in lean mass^32^.

In the current study, the correlation between BW and total fat in HFD-fed male and female mice was r = 0.854 (P < 0.01) and r = 0.988 (P < 0.01), respectively. Therefore, the significant correlation between BW change and initial BW under Oxt treatment is due to its effect on reducing fat mass.

Zhang *et al*. reported that HFD decreased plasma Oxt levels in mice^33^. One report showed elevation of plasma Oxt levels in obese subjects^34^. However, many recent studies have also reported that plasma Oxt levels are decreased in obese, type 2 diabetic, and metabolic syndrome patients^35, 36^. In addition, plasma Oxt levels negatively correlate with BMI in humans^35, 36^. However, there were no significant differences in Oxt levels between HFD-fed and standard chow-fed mice in our experiment (Fig. 1b vs Fig. 3b, male and female, respectively). This differences may be due to the difference in duration of HFD feeding period. Zhang *et al*. used mice fed with HFD for 5 month^33^. In this study, we used mice fed with HFD for only 2 month for male and 3 month for female. Therefore, it is possible that decline of plasma Oxt levels may be present only after development of severe obesity after long term HFD feeding.

In the current experiment, the plasma Oxt level was significantly or tended to be lower in male mice than in female mice. This difference of plasma Oxt levels between sex is also reported in rats and prairie voles^37^. However, no significant difference in plasma Oxt levels between males and females is reported in humans^34, 38, 39^. Further studies are required to clarify whether there is a difference in Oxt levels between sex, as seen in this study, is specific to only rodents.

However, our current results showing high plasma Oxt levels in females may suggest a reasonable explanation for the sex differences in development of obesity. It took longer periods to induce enough BW increase with HFD in female mice than male mice. Since estrogen induces Oxt gene transcription in PVN^24^, this may have led to high plasma Oxt levels in the females, which prevented fat accumulation in female mice under HFD feeding. As a result, female mice may have taken longer periods to gain weight. In this study, we evaluated the effect of Oxt by matching the initial BW as closely as possible between males and females in our study. However, it would be interesting to perform the same experiment by matching age between the male and female mice.

Reduction of plasma Oxt levels indicates important clinical implications for obesity treatment. Under obese conditions, plasma leptin and insulin levels are elevated and become resistant to leptin and insulin, contributing to further development of obesity^40, 41^. Unlike with leptin or insulin, conditions such as those in oxytocin resistance are not present in obesity. This is further supported by the fact that the effect of Oxt on BW reduction can be observed in a leptin resistant mouse model, such as db/db^42^, ob/ob^32^ mice and Zucker fatty rats^8^. It is reported that plasma oxytocinase activity is increased in liver and adipose tissues of Zucker fatty obese rats^43^. Therefore, reduced plasma oxytocin levels in obese conditions could be the result of increased peptide degradation.

The increment of Oxt receptor expression in epididymal fat was observed in leptin-resistant obese Zucker fatty rats^43^, and Oxt receptor expression in adipose tissue was negatively correlated with plasma Oxt levels^43^. These reports, which show the difference of the Oxt and Oxt receptor systems in obese conditions compared to non-obese conditions suggest that the accumulation of fat may be related to decreased plasma Oxt levels. In obese conditions, Oxt receptor expression in adipocyte is increased, thus exogenous Oxt may efficiently induce lipolysis and reduce BW.

The compensation of decreased endogenous Oxt by applying exogenesis Oxt may effectively reduce fat mass and BW in obesity. This is further supported by the dose dependent effect of exogenously applied Oxt on BW reduction in both males and females shown in this study.

In conclusion, the magnitude of BW reduction induced by Oxt correlated with initial BW and degree of fat accumulation in males and females. When treating obese patients, our study suggests that Oxt is more potent in subjects with higher BW, regardless of sex. The present study provides preclinical insight for personalized medicine to optimize the effectiveness of Oxt treatment in obese patients.

## Methods

### Animals

Male and female C57BL/6 J mice aged 6 weeks were purchased from Japan SLC (Hamamatsu, Japan). Animals were maintained on a 12 hr light/dark cycle. Dark cycle started at 19:00, and light cycle started at 07:00. The mice were fed a HFD (HFD32; Clea Osaka Japan) or standard diet (CE7: Clea Osaka Japan) for 8 weeks (males) or 12 weeks (females). Thus, 14-week-old male mice and 18-week-old female mice were used for the experiment. All experimental procedure and care of animals were carried out according to relevant guidelines and regulations and approved by Fukushima Medical University Institute of Animal Care and Use Committee.

### Measurement of food intake and BW

The initial BW of HFD-fed male mice was 37.37 ± 0.90 g for the controls and 37.34 ± 0.93 g for the Oxt group, and that of HFD-fed female mice was 33.67 ± 1.39 g for the controls and 34.7 ± 1.44 g for the Oxt group. The initial BW of standard diet-fed mice was 28.32 ± 0.52 g for the controls and 28.35 ± 0.51 g for the Oxt group, and that of standard diet-fed was 20.62 ± 0.23 g for the controls and 20.41 ± 0.28 g for the Oxt group. These HFD-fed and standard diet-fed mice were anaesthetized by intraperitoneal injection of tribromoethanol (200 mg/kg) and received surgical operation to implant osmotic mini-pumps (Alzet; model 2002, CA) into the subcutaneous tissue. Osmotic mini-pumps contained saline for the controls or Oxt (Peptide Institute, Osaka, Japan; 800 or 1600 μg/kg/day) for the Oxt group. Since the variation of BW is large in mice (especially HFD-fed mice), concentration of Oxt was calculated from individual BW, and the Oxt solution was filled into each osmotic minipump. Food and BW were measured every day at 17:00 (2 hours before the onset of the dark phase) for 10 days.

### Measurement of plasma Oxt concentration

Eight weeks HFD- or standard diet-fed male mice (14 weeks old) and 12 weeks HFD- or standard diet-fed female mice (18 weeks old) were decapitated and blood samples were collected in tubes containing EDTA and aprotinin. Plasma samples were collected between 10:30–11:30 (3.5–4.5 hours after light phase onset) under adlib fed conditions. When taking circadian rhythm into consideration, oxytocin level and secretion are high during the early light phase^44, 45^. Samples were centrifuged immediately at 4 °C at 3000 rpm for 15 min. The plasma samples were extracted by C18 Sep-Pak column (Waters MA)^46^. Plasma Oxt concentration was measured by Oxt EIA kit (Enzo Life Sciences/ Assay Designs NY)^39^. Intra-assay and inter-assay variation was 12.6–13.3% and 11.8–20.9%, respectively.

### Measurement of visceral fat and subcutaneous fat

At 10 days after implanting the mini-pump, computed tomography (CT), a La Theta LCT-200 (Hitachi Aloka Medical, Mitaka, Tokyo, Japan) was applied for measurement of visceral and subcutaneous fat mass, as described previously^47^. A Holder with a diameter of 48 mm was used. The pixel resolution was 48 μm.

Animals were scanned under isoflurane anaesthesia and the osmotic mini-pump was removed from subcutaneous fat. Animals were maintained in the CT scanner with a nose cone providing 2% isoflurane anaesthesia. Scans were done between vertebrates L1 and S4.

### Statistical analysis

All data were presented as mean ± SEM. The statistical analysis of BW change and food intake during 10 days was performed by two-way ANOVA followed by Tukey’s multiple range test. Student’s *t*-test was used for two-group comparisons. The comparisons of fat mass in the experiment using CT were analysed using a paired *t*-test. The regression coefficient was calculated to evaluate the associations between initial BW and BW change at day 10 after starting Oxt infusion in the mice. P < 0.05 was considered significant. All statistical tests were 2-tailed with 0.05 as the threshold level of significance.
